# Adverse childhood experiences are associated with increased risk of hysterectomy and bilateral oophorectomy: A national retrospective cohort study of women in England

**DOI:** 10.1111/1471-0528.17088

**Published:** 2022-02-08

**Authors:** Panayotes Demakakos, Andrew Steptoe, Gita D. Mishra

**Affiliations:** ^1^ Department of Epidemiology and Public Health University College London London UK; ^2^ Department of Behavioural Sciences and Health University College London London UK; ^3^ School of Population Health University of Queensland Brisbane Queensland Australia

**Keywords:** adverse childhood experiences, adversity, ageing, bilateral oophorectomy, cohort, epidemiology, hysterectomy, life course, observational study, retrospective study, socio‐economic position, surgical menopause

## Abstract

**Objective:**

To examine the associations between adverse childhood experiences (ACE) and the risk of hysterectomy and bilateral oophorectomy in a national sample of women in England.

**Design:**

Retrospective cohort study.

**Setting:**

A stratified random sample of households across England.

**Population:**

2648 women aged ≥55 years in 2007 from the English Longitudinal Study of Ageing (ELSA) were included in the bilateral oophorectomy analyses and 2622 in the hysterectomy analyses.

**Methods:**

Logistic and multinomial logistic regression analyses of the associations between categories of the ACE summary score (0, 1, 2, ≥3 ACE), eight individual ACE, and hysterectomy and bilateral oophorectomy.

**Results:**

615 women had undergone hysterectomy and 259 women bilateral oophorectomy. We found graded associations between the summary ACE score and risk of hysterectomy and bilateral oophorectomy. In the fully adjusted model, compared with women with no ACE, those with ≥3 ACE had double the odds of hysterectomy (odds ratio [OR] 2.01, 95% confidence interval [CI] 1.30–3.11) and more than double the odds of bilateral oophorectomy (OR 2.61, 95% CI 1.54–4.42). The exclusion of women with cancer history made the associations stronger, especially in women who underwent hysterectomy at age <40 years or bilateral oophorectomy at age ≤44 years. Several individual ACE were positively associated with both outcomes.

**Conclusions:**

ACE are associated with increased risk of hysterectomy and bilateral oophorectomy. Individual‐level covariates did not explain these associations. Our findings highlight the importance of a life course approach to understanding surgical menopause and add to our knowledge of the societal and public health impact of ACE.

**Tweetable abstract:**

Adverse childhood experiences are associated with increased risk of hysterectomy and bilateral oophorectomy in a national sample of women in England.

## Tweetable abstract

Adverse childhood experiences are associated with increased risk of hysterectomy and bilateral oophorectomy in a national sample of women in England.

**Linked article:** This article is commented on by Walter A. Rocca, pp. 1491–1492 in this issue. To view this minicommentary visit https://doi.org/10.1111/1471-0528.17105.

## 1 INTRODUCTION

Hysterectomy (the surgical removal of the uterus) is a common procedure in women of reproductive age with around 600 000 hysterectomies performed yearly in the USA.^1^ Around 90% of hysterectomies are performed to treat symptoms such as abnormal uterine bleeding and pelvic pain and benign gynaecological problems causing them, such as leiomyomas (hereafter uterine fibroids) and endometriosis.^1^, ^2^, ^3^ It has been estimated that there is an alternative treatment for >60% of these procedures and 30% of them could have been avoided.^1^, ^2^ Bilateral oophorectomy (the surgical removal of both ovaries) is often performed along with hysterectomy in the absence of ovarian indication.^4^, ^5^, ^6^, ^7^ Compared with natural menopause, surgical menopause that is caused by bilateral oophorectomy happens at younger ages and results in an abrupt termination of the ovarian production of sex steroid hormones, with major health implications.^8^, ^9^ Bilateral oophorectomy at age <45 years is associated with increased risk of cardiovascular disease,^10^, ^11^ non‐gynaecological cancers such as colorectal^12^, ^13^ and kidney cancer,^14^ osteoporosis,^8^, ^15^ and multimorbidity, accelerated ageing and worse cognitive outcomes.^16^, ^17^ Despite evidence suggesting no association,^6^, ^18^ bilateral oophorectomy at age <45 years has also been found to be associated with increased risk of mortality.^6^, ^19^, ^20^, ^21^ Hysterectomy is associated with multiple adverse health outcomes including cardiovascular disease,^22^, ^23^, ^24^ colorectal and thyroid cancer,^13^, ^14^ mental health problems^25^ and frailty.^26^

Adverse childhood experiences (ACE), a term that typically describes experiences of abuse, neglect and family disorganisation in childhood, are systematically associated with poorer physical and mental health.^27^, ^28^, ^29^, ^30^ ACE and childhood experiences of poor‐quality parenting are associated with early and late onset of menarche,^31^, ^32^, ^33^ adolescent pregnancy,^34^ menopause at younger ages,^32^ preterm delivery^35^ and miscarriage.^36^, ^37^ Importantly, ACE are associated with uterine fibroids^38^, ^39^ and endometriosis,^40^ which are common causes of hysterectomy. Within a life course perspective, ACE qualify as a causal factor associated with hysterectomy and bilateral oophorectomy, but evidence for these associations is scarce.^5^

Based on evidence suggesting an inverse association between ACE and women's health and the health‐jeopardising potential of ACE and subsequent chronic stress,^41^, ^42^, ^43^, ^44^, ^45^ we studied whether a summary ACE score was associated with increased risk of bilateral oophorectomy and hysterectomy in a national sample of women. The high prevalence of ACE, hysterectomy and surgical menopause in the population, their societal and public health impacts, and the scarcity of evidence on their association warrant our study and add value to it. To better understand the role of age and cancer in the examined associations, we conducted additional age‐stratified analyses after excluding women with cancer history. Finally, to account for ACE heterogeneity, we also examined the associations between individual ACE items and the two outcome measures.

## 2 METHODS

### 2.1 Study population

We used data from the English Longitudinal Study of Ageing (ELSA) (www.elsa‐project.ac.uk), a population‐based longitudinal study of older people. The ELSA baseline survey was carried out in 2002–2003 and involved a nationally representative sample of 11 391 individuals (6205 women) aged ≥50 years living in private addresses in England. Follow‐up surveys took place regularly every 2 years. In 2007, after the 2nd follow‐up survey (ELSA wave 3), a one‐off ELSA Life History survey took place. The aim of this survey was to collect retrospective information about the experiences and life circumstances of the participants before joining ELSA, with an emphasis on childhood and young adulthood. ACE, hysterectomy and bilateral oophorectomy were measured during the 2007 ELSA Life History survey.

Of the 4180 women who participated in the ELSA wave 3, 3441 participated in the ELSA Life History survey. The sample of the bilateral oophorectomy analysis included 2648 women aged ≥55 years in 2007 after the exclusion 518 women who did not complete the childhood experiences questionnaire, 28 who were aged ≥90 years, 120 who had their menopause at age <30 or >60 years or had missing/nonvalid menopause information, 44 who underwent bilateral oophorectomy at age <30 or >60 years or had missing/nonvalid oophorectomy information, and 83 with missing values in education and adult total net household wealth. The sample of the hysterectomy analysis included 2622 women, derived using the same exclusion criteria; however, instead of excluding women with missing/nonvalid oophorectomy information, we excluded 70 women with missing/nonvalid hysterectomy information.

ELSA has been approved by the London Multi‐Centre Research Ethics Committee (MREC/01/2/91) and informed consent has been obtained by the participants. Our study was based on secondary analysis of the ELSA data and there was no participant and public involvement.

### 2.2 Measurement of adverse childhood experiences

We defined ACE as experiences of abuse, household dysfunction and residential social care in childhood. We retrospectively measured the following eight binary ACE variables: (1) lived most of childhood in a single biological mother family, (2) lived most of childhood in social care settings (e.g. in children's home or with foster parents), (3) separation from mother for ≥6 months, (4) victim of serious physical attack/assault at age ≤16 years, (5) victim of sexual assault at age ≤16 years, (6) physically abusive parents, (7) parents with substance abuse or mental health problems and (8) parents argued or fought very often. All ACE measures refer to adversities experienced at age <16 years (unless otherwise stated).

Our study covers most of the childhood adversity domains that the original ACE study examined: abuse (physical and sexual abuse) and household dysfunction (substance abuse and mental health problems in the household, witnessing violence in the household and parental separation/divorce).^46^, ^47^ We also included a question on having spent most of the childhood in a single mother household, which is a commonly encountered childhood adversity that is related to family life and household function. In addition, we measured experience of residential social care in childhood. This is an important childhood adversity that is included in later versions of ACE measures.^48^

To make best use of all available ACE information and avoid the unnecessary exclusion of women with few missing values (the number of missing values in the eight ACE variables ranged from 61 to 3), we treated participants with missing values in the ACE variables as non‐cases, that is, we assumed that they had not experienced the missing ACE. This conservative approach resulted in analyses that were inclusive of more participants and used all ACE information available to us. Additional analyses which excluded the imputed values produced very similar results (data not shown) to those of the main analyses and thus indicated that the imputation of missing values did not bias our findings. The distribution of the eight ACE variables is presented in Tables S2 and S3. We generated a summary ACE score the way the original ACE study did.^46^, ^47^ We assigned equal weight to all eight ACE items and generated the summary ACE score by adding up all eight items. For the purposes of our analyses, we transformed this score into a categorical variable with the following categories: 0, 1, 2, ≥3 ACE. We also used the eight ACE variables as individual predictors.

### 2.3 Measurement of hysterectomy and bilateral oophorectomy

Participants were asked whether they ever had an operation to remove their uterus. Women who replied positively, were asked to report the year they had this operation. Based on this information, we generated a binary hysterectomy variable (yes/no). Because premature (at age <40 years) and early (at age 40–44 years) menopause are risk factors for multiple health problems later in life, we also generated a hysterectomy measure that combined information on both hysterectomy status and age with the following categories: no hysterectomy (reference category) and hysterectomy at age: 30–39, 40–44, 45–52 and 53–60 years.

We used the same approach to generate a binary bilateral oophorectomy variable (yes/no) and a status and age at bilateral oophorectomy variable with the following categories: no bilateral oophorectomy (reference category) and bilateral oophorectomy at age: 30–44, 45–52 and 53–60 years.

### 2.4 Covariates

To account for potential generational differences, we generated a birth cohort variable that divided participants into four birth cohorts (born between 1917 and 1926, 1927 and 1936, 1937 and 1946, and 1947 and 1952). We used this variable instead of continuous age. For a fuller exploration of the role of childhood socio‐economic position (SEP), we measured two markers: number of books in the household at age 10 years and paternal or main carer's occupational class at age 14 years. We also measured age at menarche as a marker of development and exposure to oestrogens. Regarding adult covariates, we measured several that can be on the pathway: adult SEP (education and tertiles of total net household wealth), marital status, smoking history and parity.

### 2.5 Statistical analyses

We examined the bivariate associations between the summary ACE score and covariates (Table S1). We estimated logistic regression models of the associations between the summary ACE score and the risk of hysterectomy and bilateral oophorectomy in the pooled sample (Tables 1 and 2, respectively). For each association, we computed an odds ratio (OR) and 95% confidence intervals (95% CI). In the absence of data on the indication for undergoing hysterectomy and bilateral oophorectomy and to clarify the role of gynaecological cancer in the examined associations, we performed multinomial logistic regression analyses that excluded women with history of cancer. Because we aimed to examine whether ACE were associated with hysterectomy at younger ages and premature and early surgical menopause, we performed the analyses that excluded women with cancer history were age‐stratified (Tables 3 and 4). In terms of modelling, we first estimated the unadjusted associations, which we adjusted for potential confounders: birth cohort, age at menarche and childhood SEP, and then for adult covariates: adult SEP, parity, marital status and smoking.

**TABLE 1:** The association between the summary ACE score and the risk of hysterectomy (*n* = 2622)

	Risk of hysterectomy: OR (95% CI)
Model A	
No ACE (reference category)	1.00
1 ACE	1.59 (1.19–1.95)**
2 ACEs	1.53 (1.12–2.10)*
≥3 ACEs	2.15 (1.42–3.27)**
*p*‐value for linear trend	<0.001
Model B	
No ACE (reference category)	1.00
1 ACE	1.60 (1.30–1.97)**
2 ACEs	1.46 (1.06–2.01)*
≥3 ACEs	2.06 (1.34–3.16)*
*p*‐value for linear trend	<0.001
Model C	
No ACE (reference category)	1.00
1 ACE	1.59 (1.29–1.96)**
2 ACEs	1.47 (1.07–2.03)*
≥3 ACEs	2.01 (1.30–3.11)*
*p*‐value for linear trend	<0.001

*Note*: Model A is the unadjusted association. Model B is adjusted for generation/age cohort (10‐year age cohort groups), age at menarche (≤10, 11, 12, 13, 14, 15, ≥16 years) and childhood socio‐economic position (paternal or main carer's occupational class at age 14 years and number of books in the household at age 10 years). Model C is in addition adjusted for parity (0, 1, 2, ≥3 biological children), adult socio‐economic position (education and total net household wealth), smoking history (never a smoker, ex‐smoker, current smoker) and marital status (married vs. not married).

*p ≤ 0.05, **p ≤ 0.001.

**TABLE 2:** The association between the summary ACE score and the risk of bilateral oophorectomy (*n* = 2648)

	Risk of bilateral oophorectomy: OR (95% CI)
Model A	
No ACE (reference category)	1.00
1 ACE	1.23 (0.91–1.67)
2 ACEs	1.85 (1.23–2.79)*
≥3 ACEs	2.90 (1.75–4.79)**
*p*‐value for linear trend	<0.001
Model B	
No ACE (reference category)	1.00
1 ACE	1.24 (0.91–1.68)
2 ACEs	1.75 (1.15–2.65)*
≥3 ACEs	2.82 (1.68–4.74)**
*p*‐value for linear trend	<0.001
Model C	
No ACE (reference category)	1.00
1 ACE	1.21 (0.89–1.65)
2 ACEs	1.67 (1.10–2.55)*
≥3 ACEs	2.61 (1.54–4.42)**
*p*‐value for linear trend	<0.001

*Note*: Model A is the unadjusted association. Model B is adjusted for generation/cohort category (10‐year age cohort groups), age at menarche (≤10, 11, 12, 13, 14, 15, ≥16 years) and childhood socio‐economic position (paternal or main carer's occupational class at age 14 years and number of books in the household at age 10 years). Model C is in addition adjusted for parity (0, 1, 2, ≥3 biological children), adult socio‐economic position (education and total net household wealth), smoking history (never a smoker, ex‐smoker, current smoker) and marital status (married vs. not married).

*p ≤ 0.05, **p ≤ 0.001.

**TABLE 3:** The association between the summary ACE score and the risk of hysterectomy^a^ by age at hysterectomy in women with no cancer history (*n* = 2376)

	Risk of hysterectomy at age 30–39 years: OR (95% CI)	Risk of hysterectomy at age 40–44 years: OR (95% CI)	Risk of hysterectomy at age 45–52 years: OR (95% CI)
Model A			
No ACE (reference category)	1.00	1.00	1.00
1 ACE	1.64 (1.08–2.50)*	1.55 (1.08–2.24)*	1.96 (1.42–2.72)**
2 ACEs	2.02 (1.13–3.59)*	1.34 (0.75–2.37)	1.43 (0.84–2.44)
≥3 ACEs	3.58 (1.79–7.17)**	1.84 (0.85–3.97)	2.68 (1.42–5.05)*
*p*‐value for linear trend			<0.001
Model B			
No ACE (reference category)	1.00	1.00	1.00
1 ACE	1.65 (1.08–2.53)*	1.55 (1.07–2.24)*	1.99 (1.43–2.78)**
2 ACEs	1.87 (1.05–3.37)*	1.29 (0.72–2.30)	1.38 (0.81–2.37)
≥3 ACEs	3.25 (1.53–6.65)**	1.74 (0.79–3.83)	2.72 (1.41–5.25)*
*p*‐value for linear trend			<0.001
Model C			
No ACE (reference category)	1.00	1.00	1.00
1 ACE	1.56 (1.02–2.40)*	1.58 (1.09–2.29)*	2.00 (1.43–2.78)**
2 ACEs	1.84 (1.02–3.33)*	1.35 (0.75–2.43)	1.41 (0.82–2.43)
≥3 ACEs	2.97 (1.43–6.15)*	1.63 (0.73–3.61)	2.77 (1.43–5.39)*
*p* value for linear trend			<0.001

*Note*: Model A is the unadjusted association. Model B is adjusted for generation/age cohort (10‐year age cohort groups), age at menarche (≤10, 11, 12, 13, 14, 15, ≥16 years) and childhood socio‐economic position (paternal or main carer's occupational class at age 14 years and number of books in the household at age 10 years). Model C is in addition adjusted for parity (0, 1, 2, ≥3 biological children), adult socio‐economic position (education and total net household wealth), smoking history (never a smoker, ex‐smoker, current smoker) and marital status (married vs. not married).

^a^ For the purposes of clarity, data for women who had hysterectomy at age 53–60 years are not shown.

*p ≤ 0.05, **p ≤ 0.001.

**TABLE 4:** The association between the summary ACE score and the risk of bilateral oophorectomy^a^ by age at bilateral oophorectomy in women with no cancer history (*n* = 2398)

	Risk of bilateral oophorectomy at age 30–44 years: OR (95% CI)	Risk of bilateral oophorectomy at age 45–52 years: OR (95% CI)
Model A		
No ACE (reference category)	1.00	1.00
1 ACE	1.41 (0.83–2.38)	1.16 (0.73–1.85)
2 ACEs	1.92 (0.94–3.91)	1.56 (0.82–2.97)
≥3 ACEs	3.89 (1.75–8.63)**	3.30 (1.62–6.72)**
*p*‐value for linear trend	0.001	0.003
Model B		
No ACE (reference category)	1.00	1.00
1 ACE	1.36 (0.80–2.32)	1.20 (0.75–1.92)
2 ACEs	1.73 (0.90–3.58)	1.51 (0.79–2.91)
≥3 ACEs	3.87 (1.70–8.87)**	3.31 (1.57–6.95)*
*p*‐value for linear trend	0.002	0.004
Model C		
No ACE (reference category)	1.00	1.00
1 ACE	1.32 (0.77–2.27)	1.17 (0.73–1.88)
2 ACEs	1.61 (0.77–3.37)	1.46 (0.76–2.82)
≥3 ACEs	3.48 (1.49–8.10)*	3.21 (1.51–6.82)*
*p*‐value for linear trend	0.006	0.007

*Note*: Model A is the unadjusted association. Model B is adjusted for generation/cohort category (10‐year age cohort groups), age at menarche (≤10, 11, 12, 13, 14, 15, ≥16 years) and childhood socio‐economic position (experience of severe financial crisis at age ≤16 years, paternal or main carer's occupational class at age 14 years, number of books in the household at age 10 years). Model C is in addition adjusted for parity (0, 1, 2, ≥3 biological children), adult socio‐economic position (education and total net household wealth), smoking history (never a smoker, ex‐smoker, current smoker) and marital status (married vs. not married).

^a^ For the purposes of clarity, data for women who underwent bilateral oophorectomy at age 53–60 years are not shown.

*p ≤ 0.05, **p ≤ 0.001.

To get a fuller picture of the examined associations, we also estimated models of the associations between the eight individual ACE and risk of hysterectomy and bilateral oophorectomy in the pooled sample (Tables S2 and S3). These were adjusted for childhood confounders.

## 3 RESULTS

Women with multiple ACE were more likely to be younger, of lower childhood SEP and adult wealth, current smokers, either childless or with ≥3 biological children, and reported early (≤10 years) or late menarche (≥16 years) compared with women with no ACE (Table S1).

In the pooled sample analyses, the summary ACE score was associated with increased risk of hysterectomy (Table 1) and bilateral oophorectomy (Table 2) in a graded fashion, with the latter association being stronger. In the fully adjusted model, women with ≥3 ACE had double the risk of hysterectomy (OR 2.01, 95% CI 1.30–3.11) and more than double the risk of bilateral oophorectomy (OR 2.61, 95% CI 1.54–4.42) compared with women with no ACE. The exclusion of women with cancer history made the associations stronger. The association between the summary ACE score and bilateral oophorectomy in women aged 30–44 years who had no cancer history was the strongest observed in our data (OR 3.48, 95% CI 1.49–8.10 for women with ≥3 ACE compared with those with none) (Table 4). The association between the ACE summary score and hysterectomy at age 30–39 in women with no cancer history was also strong (OR 2.97, 95% CI 1.43–6.15 for women with ≥3 ACE compared with those with none) (Table 3).

Regarding specific ACE, separation from mother for ≥6 months, parental mental health and/or substance abuse problems and very frequent parental arguments/fights (all three at age <16 years) were associated with increased risk of hysterectomy and bilateral oophorectomy in the pooled sample after adjustment for childhood confounders (Tables S2 and S3). Having lived most of childhood in a single biological mother household was associated with increased risk of hysterectomy. Physical and sexual abuse were also associated with both outcomes, but these associations did not reach statistical significance (Tables S2 and S3).

## 4 DISCUSSION

### 4.1 Main findings

In a national sample of women in England, we found that ACE were associated with increased risk of hysterectomy and bilateral oophorectomy; the greater the number of ACE, the greater the risk of both outcomes. In a similar way, the summary ACE score was associated with the risk of hysterectomy at age 30–39 years and bilateral oophorectomy at age 30–44 years in women with no cancer indication. Finally, we found that some ACE were individually associated with both outcomes.

### 4.2 Strengths and limitations

Our study is one of the first population‐based longitudinal studies on the associations between ACE and the risk of hysterectomy and bilateral oophorectomy. Unlike previous studies that used smaller regional samples,^5^, ^49^ we used data from a nationally representative sample, and this makes our findings more generalisable to community‐dwelling women. The use of data from a well‐characterised high‐quality study such as ELSA adds to the validity of our work and, along with the use of a standard set of ACE items, makes our findings more easily replicable and comparable with those of other studies. The adequate measurement of socio‐economic position over the life course allows a better understanding of the role of material deprivation and social disadvantage in the examined associations and is a strength of our study. Finally, the examination of individual ACE items next to the summary ACE score makes our work a fuller account of the association between childhood psychosocial adversity, hysterectomy and bilateral oophorectomy.

The use of retrospective data is a concern, as such data are more susceptible to measurement bias. In addition, to reduce item non‐response bias, we have recoded a few missing ACE values into negative ones. A consequence of both the retrospective measurement and recoding of missing values might be a misclassification of ACE and the inclusion of several false‐negative ACE cases in the analyses. Based on this, we assume that our findings likely underestimate the magnitude of the true associations between ACE and the two outcome measures, and are a conservative account of them. In relation to the retrospective measurement of hysterectomy, our data appear to be valid. We found that 23.5% of our participants had experienced hysterectomy, which is an estimate similar to previous estimates from both the UK and other countries.^50^, ^51^ Our retrospective childhood SEP measures have been used before and have good predictive validity. The retrospective paternal/main carer's occupation data have been found to be valid and directly comparable to prospective birth cohort data.^52^

As with most observational studies, it was impossible to eliminate confounding. Nevertheless, we were able to confirm that potentially confounding factors such as childhood SEP and age at menarche did not explain our findings. Survey non‐response is another potential source of bias. We believe that this bias has not disproportionately affected our findings, as after excluding people who died, became institutionalized or migrated, the overall individual response rate in ELSA wave 3 was 73%, 84.4% of whom participated in the ELSA Life History in 2007.^53^ Finally, we need to acknowledge the lack of statistical power in some parts of our analyses, which increased the probability of type II error and non‐significant associations. This is relevant to some of the age‐stratified analyses and analyses involving some of the individual ACE.

### 4.3 Interpretation of findings

To our knowledge, two studies have previously examined the associations between ACE and bilateral oophorectomy and hysterectomy.^5^, ^49^ Notwithstanding methodological differences, those studies reported findings similar to ours. A case–control study of 128 women who underwent bilateral oophorectomy with non‐cancer indication at age <46 years and 128 controls found individual ACE as well as a 10‐item summary ACE score to be associated with increased risk of bilateral oophorectomy.^5^ This study also found that these associations were stronger in women who had bilateral oophorectomy at age <40 years or without ovarian indication and were not explained after adjustment for education, unhealthy behaviours, obesity, reproductive history markers or marital status. Another US study of 1004 premenopausal military veterans aged ≤52 years focused exclusively on sexual abuse and found it to be associated with an increased risk of hysterectomy.^49^ In addition, two other studies reported a positive association between ACE and the risk of uterine fibroids,^38^, ^39^ a condition that is a common indication for hysterectomy and thus directly relevant to our findings. Similar associations have been reported between ACE and the risk of endometriosis,^40^ a condition that is also a common indication for hysterectomy.

There are several reasons for undergoing hysterectomy or oophorectomy. Cancer could be one such reason. Our findings, though, indicate that cancer indication did not explain the associations. The exclusion of women with cancer history from our analyses made the associations stronger, especially in younger ages.

Many women undergo hysterectomy and bilateral oophorectomy because they experience symptoms such as abnormal uterine bleeding and pain that are caused by conditions such as uterine fibroids and endometriosis. These conditions likely are on the causal pathway. We lacked data to test this hypothesis directly, but on the understanding that oestrogens are important to the development of uterine fibroids and endometriosis,^54^, ^55^ we examined age at menarche as a marker of exposure to oestrogens. We found that age at menarche explained a small part of the associations. The same applies to parity, which only explained a small part of the associations.

In the absence of data to use to examine their role, we can only speculate that ACE‐induced epigenetic alterations and chronic low‐grade inflammation, obesity and unhealthy behaviours likely are on the causal pathway. We were, however, able to examine lifetime history of smoking and found that it only explained a very small part of the associations. Another pathway that we did not examine but is relevant, is that of mental health problems.^56^

The decision to undergo hysterectomy with or without bilateral oophorectomy also involves various non‐medical factors, including patient‐, physician‐ and context‐related factors.^9^ It is reasonable to assume that patient preferences and ability to understand and play an active role in treatment decisions are possibly on the pathway. Nevertheless, our findings indicate that childhood and adult SEP including education explained a small part of the associations. These findings concur with previous findings.^5^ Childhood SEP, despite its association with ACE, appears not to be the key to explain the observed associations. This likely indicates that the effect of ACE on the risk of hysterectomy/bilateral oophorectomy has a strong psychosocial dimension. In a country with universal healthcare access such as the UK, the inability to explain the associations using individual‐level SEP markers does not preclude other contextual and systematic factors including societal, cultural and social organisation factors from partially explaining the associations. For example, previous findings suggest a positive association between perceived racism and the risk of uterine fibroids.^57^

## 5 CONCLUSION

Adverse childhood experiences are strongly associated with increased risk of hysterectomy and bilateral oophorectomy. Future research should try to determine the role of gynaecological conditions such as uterine fibroids and endometriosis in these associations and explore implicated biological pathways. Our working hypothesis is that ACE‐induced chronic stress and resultant hormonal and immune imbalances are related to the observed associations. Evidence on a positive association between stress and uterine fibroids supports this hypothesis.^58^ Mental health problems may also be relevant.^56^ On the understanding that there is a biological core in the examined associations, it is likely that our findings are relevant to and replicable in younger cohorts irrespective of the projected decline in the rate of hysterectomy.^1^ Low SEP did not explain the examined associations in our study. However, given the importance of social disadvantage for health, there is scope for more research on the direct and indirect effects of childhood social disadvantage on the examined associations.

We believe that our findings can inform clinical practice and highlight the need for a trauma‐informed approach. They can also inform and support prevention strategies.

## CONFLICT OF INTERESTS

PD reports no conflict of interest. AS holds grants from the National Institute on Aging (NIA/NIH), the National Institute for Health Research (NIHR) and the UK Research and Innovation (UKRI). GDM is the director of the Australian National Health and Medical Research Council (NHMRC) Centre for Research Excellence in women and NCD. She holds grants from the Australian Government Department of Health to conduct the Australian Longitudinal Study on Women's Health and is a senior advisor to the European Society of Menopause and Andropause. Completed disclosure of interest forms are available to view online as supporting information.

## AUTHOR CONTRIBUTIONS

PD conceived and designed the study, analysed the data, drafted the manuscript, and approved the final version to be published. AS and GDM made substantial contributions to the analysis and interpretation of the data, revised the article critically for important intellectual content article, and approved the final version to be published.

## ETHICS APPROVAL

ELSA has been approved by the London Multi‐Centre Research Ethics Committee (MREC/01/2/91) and informed consent has been obtained by the participants.

## Supporting information


Table S1–S3

## Data Availability

The data that support the findings of this study are openly available in the UK Data Service at http://doi.org/10.5255/UKDA‐SN‐5050‐23.
