# Supplementary material for: Adverse childhood experiences are associated with increased risk of hysterectomy and bilateral oophorectomy: A national retrospective cohort study of women in England
Source: BJOG. 2022 Feb 8;129(9):1481–9. doi: 10.1111/1471-0528.17088 (PMC9250543; doi:10.1111/1471-0528.17088)
Supplement: Supplementary file 1 — Table S1–S3 [file BJO-129-1481-s002.doc]

**Online Appendix S1**

**Title: Adverse childhood experiences are associated with increased risk of hysterectomy and bilateral oophorectomy: a national retrospective cohort study of women in England**

**Authors**: Panayotes DEMAKAKOS, Andrew STEPTOE, Gita D. MISHRA

| **Table S1. Sample characteristics by the summary ACE score categories (N=2,648)** | | | | | |
| --- | --- | --- | --- | --- | --- |
|  | No ACE (N=1,604) | 1 ACE (N=708) | 2 ACE (N=230) | ≥3 ACE (N=106) | *P value* |
|  | N (%) | N (%) | N (%) | N (%) | N (%) |
| **Generation/birth cohort (born between)** |  |  |  |  | 0.005 |
| 1917-1926 | 208 (13.0) | 81 (11.4) | 22 (9.6) | 3 (2.8) |  |
| 1927-1936 | 438 (27.3) | 236 (33.3) | 64 (27.8) | 26 (24.5) |  |
| 1937-1946 | 539 (37.0) | 234 (33.1) | 92 (40.0) | 46 (43.4) |  |
| 1947-1952 | 365 (22.7) | 157 (21.2) | 52 (22.6) | 31 (29.3) |  |
| **Paternal or main carer’s occupation at age 14 years** |  |  |  |  | 0.004 |
| Managerial, Professional, Business owner, Administration | 558 (34.8) | 217 (30.6) | 68 (30.7) | 23 (21.7) |  |
| Sales, trade and care professions | 496 (30.9) | 202 (28.5) | 65 (28.6) | 26 (24.5) |  |
| Plant worker, causal jobs, unemployed | 419 (30.6) | 256 (36.2) | 86 (36.1) | 45 (42.5) |  |
| Other incl. pensioners, armed forces and missing* | 59 (3.7) | 33 (4.7) | 11 (4.6) | 12 (11.3) |  |
| **Number of books in the household at age 10 years** |  |  |  |  | <0.001 |
| Enough to fill ≥two bookcases (>100) | 314 (19.6) | 132 (18.6) | 33 (14.4) | 18 (17.0) |  |
| Enough to fill one bookcase (26-100) | 516 (32.2) | 191 (27.0) | 54 (23.5) | 18 (17.0) |  |
| Enough to fill one shelf (11-25) | 396 (24.7) | 157 (22.2) | 56 (24.3) | 20 (18.9) |  |
| None or very few | 331 (20.6) | 189 (26.7) | 69 (30.0) | 35 (33.0) |  |
| Missing/other* | 47 (2.9) | 39 (5.5) | 18 (7.8) | 15 (14.1) |  |
| **Education** |  |  |  |  | 0.15 |
| A-level or higher | 442 (27.5) | 179 (25.3) | 53 (23.0) | 36 (34.0) |  |
| GCSE, O-level or equivalent | 558 (34.8) | 239 (33.7) | 75 (32.6) | 28 (26.4) |  |
| No educational qualifications | 604 (37.7) | 290 (41.0) | 102 (44.4) | 42 (39.6) |  |
| **Tertiles of total net non-pension household wealth** |  |  |  |  | 0.034 |
| Wealthiest tertile (> £295,801) | 554 (34.5) | 231 (32.6) | 71 (31.9) | 29 (27.4) |  |
| Intermediate tertile (≤ £295,800 to ≥£152,600) | 544 (33.9) | 228 (32.2) | 85 (36.9) | 26 (24.5) |  |
| Poorest tertile (<£152,600) | 506 (31.6) | 249 (35.2) | 74 (32.2) | 51 (48.1) |  |
| **Married** |  |  |  |  | 0.54 |
| No | 566 (35.3) | 258 (36.4) | 78 (33.9) | 44 (41.5) |  |
| Yes | 1,038 (64.7) | 450 (63.6) | 152 (66.1) | 62 (58.5) |  |
| **Smoking history** |  |  |  |  | <0.001 |
| Never a smoker | 786 (49.0) | 305 (43.1) | 84 (36.5) | 35 (33.0) |  |
| Ex-smoker | 592 (36.9) | 288 (40.7) | 101 (43.9) | 42 (39.6) |  |
| Current smoker | 226 (14.1) | 115 (16.2) | 45 (19.6) | 29 (27.4) |  |
| **Age at menarche (in years of age)** |  |  |  |  | 0.026 |
| ≤10 | 70 (4.4) | 33 (4.7) | 17 (7.4) | 10 (9.4) |  |
| 11 | 258 (16.1) | 121 (17.1) | 56 (23.4) | 15 (14.2) |  |
| 12 | 249 (15.5) | 107 (15.1) | 30 (13.0) | 17 (16.1) |  |
| 13 | 375 (23.4) | 150 (21.2) | 40 (17.4) | 21 (19.8) |  |
| 14 | 336 (20.9) | 163 (23.0) | 49 (21.3) | 19 (17.9) |  |
| 15 | 204 (12.7) | 81 (11.4) | 25 (10.9) | 10 (9.4) |  |
| ≥16 | 112 (7.0) | 53 (7.5) | 13 (5.6) | 14 (13.2) |  |
| **Parity (number of biological children)** |  |  |  |  | 0.018 |
| 0 | 189 (11.8) | 91 (12.9) | 34 (14.8) | 16 (15.1) |  |
| 1 | 206 (12.8) | 90 (12.7) | 22 (9.6) | 8 (7.5) |  |
| 2 | 678 (42.3) | 250 (35.3) | 87 (37.8) | 37 (34.9) |  |
| ≥3 | 531 (33.1) | 277 (39.1) | 87 (37.8) | 45 (43.5) |  |
| **Age at bilateral oophorectomy (categories)** |  |  |  |  | 0.001 |
| No bilateral oophorectomy | 1,471 (91.7) | 637 (90.0) | 197 (86.6) | 84 (79.2) |  |
| Yes, at age 30-44 years | 50 (3.1) | 29 (4.1) | 11 (4.8) | 9 (8.5) |  |
| Yes, at age 45-52 years | 61 (3.8) | 31 (4.4) | 14 (6.1) | 11 (10.4) |  |
| Yes, at age 53-60 years | 22 (1.4) | 11 (1.5) | 8 (3.5) | 2 (1.9) |  |
| **Age at hysterectomy (categories)**** |  |  |  |  | <0.001 |
| No hysterectomy | 1,275 (80.1) | 498 (71.6) | 165 (72.4) | 69 (65.1) |  |
| Yes, at age 30-39 years | 76 (4.8) | 40 (5.8) | 19 (8.3) | 11 (10.4) |  |
| Yes, at age 40-44 years | 95 (5.9) | 60 (8.6) | 16 (7.0) | 10 (9.4) |  |
| Yes, at age 45-52 years | 104 (6.5) | 80 (11.5) | 20 (8.8) | 15 (14.2) |  |
| Yes, at age 53-60 years | 43 (2.7) | 17 (2.5) | 8 (3.5) | 1 (0.9) |  |
| *Other/missing category has not been used in the calculation of the *P value*  ***The sample for the hysterectomy analysis was 2,622*  *P values were generated using Analysis of Variance (ANOVA), Kruskal-Wallis and chi square tests for continuous, ordinal and categorical covariates, respectively.* | | | | | |

| **Table S2. The associations between individual ACE and the risk of hysterectomy (N=2,622)** | |
| --- | --- |
|  | Risk of hysterectomy: OR (95% CI) |
| Sexual assault at age ≤16 years |  |
| No/missing (reference category) (n=2,510) | 1.00 |
| Yes (n=112) | 1.43 (0.94 to 2.19) |
| Physical attack/assault at age ≤16 years |  |
| No/missing (reference category) (n=2,596) | 1.00 |
| Yes (n=26) | 1.69 (0.74 to 3.86) |
| Physically abusive parents at age <16 years |  |
| No/missing (reference category) (n=2,535) | 1.00 |
| Yes (n=87) | 0.84 (0.50 το 1.43) |
| Spent most of childhood in a single biological mother household |  |
| No/missing (reference category) (n=2,476) | 1.00 |
| Yes (n=146) | 1.45 (1.00 to 2.11)* |
| Spent most of childhood in children’s home or with foster parents |  |
| No/missing (reference category) (n=2,543) | 1.00 |
| Yes (n=79) | 0.68 (0.38 to 1.21) |
| Separation from mother for ≥6 months at age <16 years |  |
| No/missing (reference category) (n=2,220) | 1.00 |
| Yes (n=402) | 1.60 (1.25 to 2.04)** |
| Parental mental health or substance abuse problems at age <16 years |  |
| No/missing (reference category) (n=2,470) | 1.00 |
| Yes (n=152) | 1.74 (1.22 to 2.47)* |
| Parents argued/fought very often when respondent aged <16 years |  |
| No/missing (reference category) (n=2,108) | 1.00 |
| Yes (n=514) | 1.33 (1.07 to 1.67) |
| All models are adjusted for generation/cohort category (10-year age cohort groups), age at menarche (≤10, 11, 12, 13, 14, 15, ≥16 years), and childhood socioeconomic position (severe paternal or main carer’s occupational class at age 14 years and number of books in the household at age 10 years)  ^*^*P ≤*0.05, ^**^*P ≤*0.001 | |

| **Table S3. The associations between individual ACE and the risk of bilateral oophorectomy (N=2,648)** | |
| --- | --- |
|  | Risk of bilateral oophorectomy: OR (95% CI) |
| Sexual assault at age ≤16 years |  |
| No/missing (reference category) (n=2,536) | 1.00 |
| Yes (n=112) | 1.56 (0.89 to 2.74) |
| Physical attack/assault at age ≤16 years |  |
| No/missing (reference category) (n=2,623) | 1.00 |
| Yes(n=25) | 1.86 (0.62 to 5.62) |
| Physically abusive parents at age <16 years |  |
| No/missing (reference category) (n=2,562) | 1.00 |
| Yes (n=86) | 0.99 (0.49 το 2.02) |
| Spent most of childhood in a single biological mother household |  |
| No/missing (reference category) (n=2,497) | 1.00 |
| Yes (n=151) | 1.51 (0.92 to 2.48) |
| Spent most of childhood in children’s home or with foster parents |  |
| No/missing (reference category) (n=2,568) | 1.00 |
| Yes (n=80) | 0.83 (0.37 to 1.85) |
| Separation from mother for ≥6 months at age <16 years |  |
| No/missing (reference category) (n=2,237) | 1.00 |
| Yes (n=411) | 1.84 (1.33 to 2.55)** |
| Parental mental health or substance abuse problems at age <16 years |  |
| No/missing (reference category) (n=2,494) | 1.00 |
| Yes (n=154) | 1.98 (1.28 to 3.07)* |
| Parents argued/fought very often when respondent aged <16 years |  |
| No/missing (reference category) (n=2,132) | 1.00 |
| Yes (n=516) | 1.42 (1.04 to 1.92)* |
| All models are adjusted for generation/cohort category (10-year age cohort groups), age at menarche (≤10, 11, 12, 13, 14, 15, ≥16 years), and childhood socioeconomic position (severe paternal or main carer’s occupational class at age 14 years and number of books in the household at age 10 years)  ^*^*P ≤*0.05, ^**^*P ≤*0.001 | |
